# The Evolving Role of Ferroptosis in Breast Cancer: Translational Implications Present and Future

**DOI:** 10.3390/cancers13184576

**Published:** 2021-09-12

**Authors:** Hung-Yu Lin, Hui-Wen Ho, Yen-Hsiang Chang, Chun-Jui Wei, Pei-Yi Chu

**Affiliations:** 1 Graduate Institute of Biomedical Engineering, National Chung Hsing University, Taichung 402, Taiwan; linhungyu700218@gmail.com; 2 Research Assistant Center, Show Chwan Memorial Hospital, Changhua 500, Taiwan; wen9805@gmail.com; 3 Department of Nuclear Medicine, Kaohsiung Chang Gung Memorial Hospital and Chang Gung University College of Medicine, Kaohsiung 833, Taiwan; changyh@cgmh.org.tw; 4 Center for Mitochondrial Research and Medicine, Kaohsiung Chang Gung Memorial Hospital, Kaohsiung 833, Taiwan; 5 Department of Pathology, Show Chwan Memorial Hospital, Changhua 500, Taiwan; 6 School of Medicine, College of Medicine, Fu Jen Catholic University, New Taipei City 242, Taiwan; 7 Department of Health Food, Chung Chou University of Science and Technology, Changhua 510, Taiwan; 8 National Institute of Cancer Research, National Health Research Institutes, Tainan 704, Taiwan

**Keywords:** breast cancer, therapy, ferroptosis, iron metabolism, lipid peroxidation, antioxidant defense

## Abstract

**Simple Summary:**

Despite decades of extensive study into targeting cell death in breast cancer, including apoptosis, the clinical treatment remains challenging due to its high probability of recurrence. As an emerging form of cell death, ferroptosis features suppression of drug resistance and augmentation of antitumor immunity. The advances in the development of clinical drugs targeting ferroptosis provide growing silver linings for breast cancer treatment. Research into biomarkers to precisely trace ferroptosis in patients with cancer, and the development and subsequent application of novel ferroptosis-based therapies will be of critical importance in the next few years.

**Abstract:**

Breast cancer (BC) is the most common malignancy among women worldwide. The discovery of regulated cell death processes has enabled advances in the treatment of BC. In the past decade, ferroptosis, a new form of iron-dependent regulated cell death caused by excessive lipid peroxidation has been implicated in the development and therapeutic responses of BC. Intriguingly, the induction of ferroptosis acts to suppress conventional therapy-resistant cells, and to potentiate the effects of immunotherapy. As such, pharmacological or genetic modulation targeting ferroptosis holds great potential for the treatment of drug-resistant cancers. In this review, we present a critical analysis of the current understanding of the molecular mechanisms and regulatory networks involved in ferroptosis, the potential physiological functions of ferroptosis in tumor suppression, its potential in therapeutic targeting, and explore recent advances in the development of therapeutic strategies for BC.

## 1. Introduction

Breast cancer (BC) is the most commonly diagnosed cancer among women, and is the fourth leading cause of cancer deaths worldwide, according to a status report on the global cancer burden provided by GLOBOCAN 2020 [1]. To date, the standard treatments for patients with BC include surgery, radiation therapy, hormone therapy, and chemotherapy [2,3]. The cause of death in patients with BC is primarily related to cancer metastasis and relapse, which are associated with metabolic reprogramming that fosters a corrupted tumor microenvironment (TME) to counteract therapy-induced cell death [4]. Regulated cell death (RCD) is an autonomous and orderly death. In addition to apoptosis and necroptosis, recent studies have revealed new modes of RCD, including pyroptosis and ferroptosis [5,6,7,8]. All of these death modes present distinct features in terms of cellular morphology, biochemistry, and signaling pathways (Table 1). Despite decades of extensive study into targeting cancer cell death, such as approaches targeting caspases and BCL-2 families in apoptosis, the clinical implementation of related therapeutic agents remains challenging [9]. Indeed, cancer cells present resistance against apoptotic cellular death [10]. Therefore, targeting a nonapoptotic RCD may offer an alternative path to the development of effective cancer therapeutics.

Apoptosis can be triggered by extrinsic (also known as death receptor-activated) and intrinsic (also known as mitochondrial or BCL-2 regulated) pathways. The extrinsic pathway can be activated by the ligation of tumor necrosis factor receptor (TNFR) superfamily members, which promotes adaptor proteins (e.g., FADD) to activate caspase-8 and then the downstream effector caspase-3 and -7 [11]. The intrinsic pathway can be induced by intrinsic stress (growth factor deprivation, DNA damage, and endoplasmic reticulum stress), and BH3-only proteins (PUMA, NOXA, BIM, BID, BAD) [12,13]. For example, p53-upregulated PUMA can bind with a high affinity to BCL-2, thereby liberating BAX/BAK to the mitochondria. This results in the formation of mitochondrial outer membrane permeabilization (MOMP) and the released cytochrome c binding to APAF-1 to form an apoptosome, leading to apoptosis. Under the induction of endoplasmic reticulum stress, the conformational activation of BAX/BAK acts at the mitochondrial membrane, thereby relaying the signaling for the assembly of the apoptosome [14]. In necroptosis, tumor necrosis factor α (TNFα), the CD95 receptor/Fas ligand complex, and other members of the TNF superfamily were identified as inducers [15]. Receptor-interacting protein kinase 1 (RIPK1), RIPK3 and the mixed lineage kinase domain-like pseudokinase (MLKL) are required proteins for the activation of necroptosis. In response to death receptor activation, the binding of RIPK1 to RIPK3 triggers the formation of necrosomes, resulting in MLKL activation [8]. As a necroptotic effector, the activated MLKL translocates to the plasma membrane, causing permeabilization and subsequent cell death. The predominant hallmarks of pyroptosis are the activation of an inflammasome, a cytosolic multiprotein complex accounting for the release of interleukin-1β (IL-1β) and IL-18, the formation of an apoptosis-associated speck-like protein containing a CARD (ASC), and the activation of proinflammatory cascades [16]. Generally, pattern recognition receptors (PRRs, e.g., nod-like receptor 3 (NLRP3) and absent in melanoma-like receptor 2 (AIM2)) first recognize a variety of dangerous signals, then activate procaspase-1 cleavage and ASC recruitment to assemble inflammasomes. Activated caspase-1 acts to cleave the pyroptosis executor gasdemin D (GSDMD) at the Asp275 site to free the N-terminal domain (GSDMD-NT) and generate nonselective pores on the cell membrane. Meanwhile, caspase-1 cleaves and activates the precursors of IL-1β and IL-18 to produce mature IL-1β and IL-18. The intracellular contents are then released through pores caused by GSDMD-NT, leading to pyroptosis [16]. In addition, an inflammasome-independent, non-canonical pathway mediated by a caspase-1/4/5/11-cleaved GSDMD-NT was recognized [17,18,19]. Furthermore, caspase-3, an iconic apoptosis-related caspase, was revealed to trigger gasdemin E (GSDME)-dependent pyroptosis under the scenario of chemotherapy drug treatment [20,21]. Ferroptosis is characterized by an iron-dependent manner of RCD, described in detail below. Table 1 summarizes the basic features and representative signaling molecules of the four RCDs.

In terms of anticancer immunity, apoptosis has mainly been considered as tolerogenic cell death (TCD), while some reports suggested that apoptosis is involved in immunogenic cell death (ICD) [15,22]. In contrast, mounting evidence has revealed that ICD can be mediated by the activation of ferroptosis, necroptosis, and pyroptosis [22]. The existence and clarification of the interconnectivity of various RCDs renders growing silver linings for the development of anticancer therapeutics. For example, signatures of pro-ferroptosis, pro-necroptosis, and pro-pyroptosis were reported to be associated with CD8+ T cell infiltration across seven common cancers [22]. The sensitivity of the genes involved in ferroptosis, necroptosis and pyroptosis were shown to be positively correlated with microsatellite instability (MSI) and tumor mutation burden (TMB) in a portion of cancers [22]. Therapeutically, clinically approved antitumor drugs that target immunity are reported to induce ICD by way of combined RCDs. For instance, artesunate was shown to simultaneously elicit ferroptosis and necroptosis in cancer cells [23] and exert a potentiation effect on antitumor immunity [24,25,26]. Doxorubicin was demonstrated to enhance antitumor immunity through the induction of both ferroptosis and pyroptosis. In a mechanistic regard, necroptosis acts to interconnectedly facilitate pyroptosis via activation of the receptor-interacting protein kinase 1 (RIPK1) pathway, which then activates the Nod-like receptor 3 (NLRP3)-caspase-1 pathway [27]. The concomitant induction of ferroptosis and pyroptosis was reported to be mediated by the perturbation of glutathione (GSH) and the activation of gasdermin E (GSDME) in the context of cisplatin exposure [28,29].

In 2012, Dixon et al. first described the concept of ferroptosis [30], which refers to an iron-dependent cell death caused by lipid peroxidation and subsequent plasma membrane rupture [31]. Differing from other types of RCD, ferroptosis does not present the cellular swelling observed in necroptosis and pyroptosis, nor the cellular shrinkage and formation of apoptotic bodies exhibited in apoptosis (Table 1). In terms of organellar morphology, ferroptosis does not exhibit chromatin condensation in the nucleus or cytoskeletal disintegration; it does, however, manifest a distinct disorganization of mitochondria including mitochondrial shrinkage, vanishing of mitochondrial cristae, and rupture of the outer mitochondrial membrane (OMM). A growing body of study has led to the identification of an intricate signaling pathway that controls ferroptosis by inducing iron accumulation and lipid peroxidation or perturbing the antioxidant defensive system. Importantly, recent reports have revealed that cancer cells which are resistant to conventional therapy or harbor a propensity to metastasize are vulnerable to ferroptosis, and that immunotherapeutic effects can be potentiated by ferroptosis [32,33,34,35]. In light of this, we herein aimed to review the latest research on ferroptosis to further the understanding of its pathogenesis and to propose new targets for the treatment of BC (Table 2).

**Table 1:** Characteristics of ferroptosis, apoptosis, necroptosis, and pyroptosis.

Characteristics	Ferroptosis	Apoptosis	Necroptosis	Pyroptosis
Morphological features	small mitochondria,	unaltered mitochondria	swollen mitochondria	unaltered mitochondria
	vanishing mitochondrial cristae	apoptotic bodies	release of cytoplasmic constituents	Pore formation on plasma membrane
	OMM rupture	cytoskeletal disintegration	plasma membrane rupture	Inflammasome formation
	normal nucleus	chromatin condensation	chromatin condensation	Chromatin condensation
	normal cell size	shrinkage of cell	swollen cell	swollen cell
Biochemical hallmarks	iron accumulation	PS exposure	No PS exposure *	secretion of IL-18 and IL-1β
	lipid peroxidation	DNA fragmentation	depletion of ATP	
Primary immune features	ICD	TCD	ICD	ICD
Signaling pathways	TFRC IREB/SLC11A2	TNFRSF1A/FADD TRAILR1/2 Caspases-9/-3/-7 P53/PUMA BCL-2/BAX/BAK cytochrome c/APAF-1 endoplasmic reticulum	TNFR1/RIPK1 RIPK1/RIPK3/MLKL PKC-MAPK-AP-1	release of mtDNA in cytosol NLRP3/AIM2 caspase-1/ IL-18/IL-1β capase-1/4/5/11/GSDMD caspase-3/ GSDME
	Haem/HO-1 FTH1/FTL/Prominin 2 NCOA4/ferrotinophagy CISD1/2/Fe-S ACSLs/LPCAT3/ALOXs			
	GSH/GPX4, MVA/HMGCR			
	FSP1/CoQ10 DHODH/CoQ10			

AIM2, absent in melanoma like receptor 2; AP-1, activator protein 1; APAF-1, apoptotic peptidase activating factor 1; BAK, Bcl-2 homologous antagonist/killer; BAX, Bcl-2-associated X; BCL2, B-cell lymphoma 2; FADD, Fas-associated protein with death domain; GSDMD, gasdermin D; GSDME, gasdermin E; ICD, immunogenic cell death; MAPK, mitogen-activated protein kinase; MLKL, mixed lineage kinase domain-like pseudokinase; NLRP3, Nod-like receptor 3; OMM, outer membrane of mitochondria; PKC, protein kinase C; PS, phosphatidylserine; PUMA, p53 upregulated modulator of apoptosis; RIPK1, receptor-interacting protein kinase 1; RIPK3, receptor-interacting protein kinase 3; TCD, tolerogenic cell death; TNFR1, tumor necrosis factor receptor 1; TNFRSF1A, tumor necrosis factor receptor superfamily member 1A; TRAILR, tumor necrosis factor-related apoptosis-inducing ligand receptor-1. * While Annexin V assay remains the most widely used assay for apoptosis detection based on PS exposure, several reports identified this phenomenon in necroptosis as well. Wang et al., in this regard, demonstrated a necroptotic phenotype with an increased PS exposure proportion, activated RIPK1/MLKL axis, and damaged plasma membrane [36].

**Table 2:** Updated therapeutic approaches for targeting of ferroptotic pathways in BC.

Target	Approaches	Phase of Clinical Development	Reference
**Iron activators**			
↑Transferrin	lapatinib	in vitro model	28827805 [37]
↑Transferrin	lapatinib	in vitro model	27441659 [38]
↓SLC40A1	lapatinib	Marketed	NCT00667251 [38]
↑Iron	UV light	Preclinical animal model	32804509 [39]
↑Iron	MOF-Fe^2+^	Preclinical animal model	32944722 [40]
↑Iron	Fe_3_O_4_ nanocapcules	in vitro model	34225869 [41]
↑Iron	neratinib	Preclinical animal model	31409375 [42]
↑Iron	neratinib	Marketed	NCT04366713
↑Iron	neratinib	Marketed	NCT03377387
↑Iron	Fe^3+^-PDA NP	in vitro model	33808898 [43]
↓CISD1/2, ↑Iron	MAD-28	Preclinical animal model	25762074 [44]
↑HO-1	MI-463	in vitro model	32945449 [45]
↑HO-1	SGNI	Preclinical animal model	33827043 [46]
**Ferritinophagy activators**			
↑Iron	artesunate	Phase I trials	NCT00764036 [47]
**Lipid peroxides activators**			
↑ACSL1	α-eleostearic acid	Preclinical animal model	33854057 [48]
↑ACSL4	Polyphyllin III	Preclinical animal model	34040532 [49]
**System xc- inhibitors**			
↓SLC7A11	Erastin	in vitro model	33672555 [50]
↓SLC7A11	Lidocaine	in vitro model	34122108 [51]
↓SLC7A11	miR-106a-5p	in vitro model	33686957 [52]
↓SLC7A11	sulfasalazine	Phase I trials	NCT03847311
↓SLC7A11	metformin, sulfasalazine	in vitro model	34162423 [53]
↓SLC7A11	18-β-glycyrrhetinic acid	in vitro model	34271106 [54]
**GPX4 pathway inhibitors**			
↓GPX4	JQ1+BTZ	Preclinical animal model	32937365 [55]
↓NRF2	overexpression of GSK-3β	Preclinical animal model	32642794 [56]
↓GPX4	RSL-3	Preclinical animal model	34170581 [57]
↓GPX4	metformin	Preclinical animal model	33522578 [58]
↓GPX4	DMOCPTL	Preclinical animal model	33472669 [59]
↓GPX4, MDM2	Compound 3d	Preclinical animal model	33725632 [60]
↓TYRO3	LDC1267	Preclinical animal model	33855973 [32]
↓DHODH	leflunomide	Preclinical animal model	32034120 [61]
↓DHODH	BQR, leflunomide, 4SC-101	in vitro model	28196676 [62]
**HMGCR inhibitor**			
↓HMGCR	fluvastatin	Phase I trials	19728082 [63]
↓HMCGR	atorvastatin	Phase II trials	NCT00816244 [64]

↑, promote; ↓, inhibit; ACSL1/4, Acyl-CoA synthetase long chain family members; BQR, brequinar sodium; CISD1/2, CDGSH iron sulfur domain 1/2; DHODH, dihydroorotate dehydrogenase; GPX4, glutathione peroxidase 4; GSK-3β, glycogen synthase kinase 3 beta; HMGCR, HMG-CoA reductase; HO-1, heme oxygenase-1; MDM2, mouse double minute 2; MOF-Fe^2+^, metal-organic framework-based Fe^2+^ delivery; MI-463, menin-mixed-lineage leukemia (MLL) inhibitor-463; NRF2, nuclear factor, erythroid 2-like 2; PDA NPs, polydopamine nanoparticles; SGNI, Shuganning injection; SLC40A1, solute carrier family 40 member 1; SLC7A11, solute carrier family 7 member 11; TYRO3, tyrosine protein kinase receptor 3.

## 2. Inducing Ferroptosis by Iron Toxicity and Lipid Peroxides

**Figure 1:**
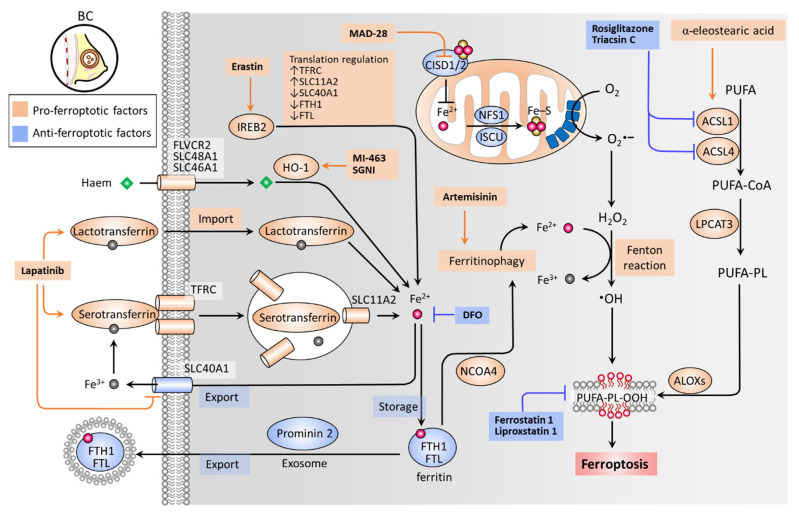
Iron metabolism and lipid peroxides in ferroptosis. Ferroptosis is primarily triggered by iron accumulation-mediated lipid peroxidation, which is determined by key factors contributing to the import, export, and metabolism of iron and the formation of PUFA-PL-OOH. Serotransferrin-TFRC complexes load iron and facilitate its import through endosome internalization, by which iron can be released via SLC11A2. IREB2 transcriptional activity, heme/HO-1, and lactotransferrin provide additional routes of intracellular iron import. DFO acts as a scavenger of intracellular iron. FTH1 and FTL together form an iron storage protein complex (ferritin), preventing Fe^2+^ from being oxidized to Fe^3+^ by the Fenton reaction. Prominin-2 acts to form FTH1/FTL/iron complex-containing exosomes, which are then exported to the extracellular space. NCOA4 plays a role in inducing autophagic degradation of FTH1/FTL (ferritinophagy), leading to an increase in intracellular iron. ACSLs and LPCAT3 facilitate the formation of PUFA-PLs, which are susceptible to •OH-catalyzed oxidation that is mediated by ALOXs. Mitochondria serve as a main source of intracellular superoxide (O_2_•^−^), which can be reduced to H_2_O_2_ by dismutase. The partially reduced H_2_O_2_ by iron generates •OH, a process called the Fenton reaction. •OH can act to abstract hydrogen atoms from PUFA-PLs to activate lipid peroxidation and the accumulation of PUFA-PL-OOH, ultimately causing ferroptosis. •OH, hydroxyl radical; ACSL, acyl-CoA synthetase long chain family members; ALOXs, lipoxygenases; CISD1/2, CDGSH iron sulfur domain 1/2; DFO, desferoxamine; FTH1, ferritin heavy chain 1; FTL, ferritin light chain; H_2_O_2_, hydrogen peroxide; HO-1, heme oxygenase-1; IREB2, iron response element binding protein 2; ISCU, iron-sulfur cluster assembly enzyme; LPCAT3, lysophospholipid acyl-transferase 3; NCOA4, nuclear receptor co-activator 4; NFS1, NFS1 cysteine desulfurase; PUFA-PL-OOH, lipid peroxides generated from polyunsaturated fatty acid-containing phospholipids; SGNI, Shuganning injection; SLC11A2, solute carrier family 11 member 2; SLC40A1, solute carrier family 40 member 1; TFRC, transferrin receptor.

Iron accumulation and lipid peroxidation are two key hallmarks of ferroptosis [30]. Iron is an important trace element, while an aberrant distribution or content of iron in the body can lead to physiological disorders. Iron imported into a cell can be mediated by serotransferrin through the transferrin receptor (TFRC) (Figure 1) [65]. Iron-loaded serotransferrin-TFRC complexes are internalized through endosomes, where they release iron (Fe^2+^) into the cytoplasm through solute carrier family 11 member 2 (SLC11A2), leading to increased iron accumulation and subsequent induction of ferroptosis [65] (Figure 1). Lactotransferrin and heme provide additional sources of iron through differing import mechanisms in the cell membrane [31]. On the other hand, iron export mediated by solute carrier family 40 member 1 (SLC40A1) inhibits ferroptosis [66]. Knockdown of TFRC can inhibit erastin-induced ferroptosis [67], while heme oxygenase-1 (HO-1) can accelerate erastin-induced ferroptosis by supplementing iron [68]. Ferritin acts as an iron storage protein complex, which is composed of ferritin heavy chain 1 (FTH1) and ferritin light chain (FTL) [69]. Brown et al. reported that prominin-2 acts to form ferritin-containing exosomes, which are exported out of the cell to prevent ferroptosis [70]. As a transcription factor, iron response element binding protein 2 (IREB2) acts to mediate the pro-ferroptosis effect of erastin by increasing expression levels of TFRC and SLC11A2, while decreasing those of SLC40A1, FTH1, and FTL [71]. Ferritinophagy (an autophagic degradation of ferritin), mediated by nuclear receptor coactivator 4 (NCOA4), can enhance intracellular iron (Fe^2+^) levels and ultimately result in ferroptosis [72,73] (Figure 1). In mitochondria, proteins involved in the utilization of iron for iron-sulfur cluster biogenesis, including cysteine desulfurase (NFS1), iron-sulfur cluster assembly enzyme (ISCU), CDGSH iron sulfur domain 1 (CISD1, also known as mitoNEET), and CISD2 (also known as nutrient-deprivation autophagy factor-1 (NAF-1)), inhibit ferroptosis by increasing the biosynthesis of iron-sulfur clusters (Fe-S), thereby reducing intracellular iron levels [74,75,76,77]. Intracellular iron excess can promote subsequent lipid peroxidation by way of at least two mechanisms: (1) the iron-dependent Fenton reaction that produces reactive oxygen species (ROS); and (2) the activation of iron-containing enzymes such as lipoxygenases (ALOXs) [31,78,79]. In the process of ferroptosis, polyunsaturated fatty acids (PUFAs) are most susceptible to lipid peroxidation, which can lead to a damaged membrane structure [80]. Acyl-CoA synthetase long chain family members (ACSLs) and lysophospholipid acyltransferase 3 (LPCAT3) promote the incorporation of polyunsaturated fatty acids (PUFAs) into phospholipids (PLs) to form polyunsaturated fatty acid-containing phospholipids (PUFA-PLs), which are sensitive to ROS-initiated oxidation mediated by ALOXs, leading to the formation of lipid peroxides (PUFA-PL-OOH), and ultimately ferroptosis [81,82,83,84] (Figure 1). 

Zhu et al. have reported that irradiation-activated azobenzene combretastatin A4 (Azo-CA4)-loaded nanocarriers promote the ferroptosis of TNBC cells [39]. A UV light-triggered reduction of Fe^3+^ to Fe^2+^ induces ferroptosis, while the photoisomerization of Azo-CA4 elicits apoptosis. Xu et al. designed a metal–organic framework (MOF)-based Fe^2+^ delivery system to increase intracellular iron toxicity, whereby BC cells and in vivo tumors undergo ferroptosis [40]. Nieto et al. revealed that Fe^3+^ loaded-polydopamine nanoparticles induce ferroptosis by increasing intracellular levels of iron, exerting a synergetic effect on the combination of doxorubicin [43]. Antoniak et al. demonstrated that nanocapsules containing Fe_3_O_4_ induce BC cell ferroptosis [41]. Notably, TNBC (MDA-MB-231) cells were more susceptible to Fe_3_O_4_ nanocapsules than estrogen receptor (ER)-positive (MCF7) cells. Kato et al. reported that menin-mixed-lineage leukemia inhibitor MI-463 induces ferroptosis in an HO-1 activity-dependent manner [45]. Du et al. have shown that Shuganning injection (SGNI), a traditional Chinese patent medicine, can induce ferroptosis and in vivo tumor growth in TNBC cells by inducing HO-1, which acts to promote intracellular iron accumulation [46]. Ma et al. reported that lapatinib promotes the accumulation of intracellular iron, ROS expression levels, and ultimately ferroptosis by increasing expression levels of transferrin and decreasing SLC40A1 (also known as ferroportin-1) [37,38]. Nagpal et al. revealed that neratinib induces ferroptosis by increasing intracellular iron levels [42]. Induced ferritinophagy has been reported to mediate ferroptosis and the antitumor effect of artemisinins [85,86,87], which are derived from the Chinese herb *Artemisia annua*, categorized as a class of antimalarial drugs [88]. An artemisinin derivative, artesunate, was demonstrated as being safe and well-tolerated at an oral dose of up to 200 mg when applied to patients with metastatic BC (NCT00764036; completed phase I clinical trial) [47]. Bai et al. revealed that a derivative of mitocan (small molecules selectively targeting mitochondria), MAD-28, acts to promote ferroptosis by inhibiting CISD1 and CISD2, and increasing mitochondrial iron levels [44]. Zhou et al. demonstrated that Polyphyllin III, a major saponin extracted from *Paris polyphylla* rhizomes, promotes ferroptosis of TNBC cells through ACSL4-mediated lipid peroxidation [49]. The combination of Polyphyllin III and sulfasalazine (an inhibitor of system xc-) exhibited a potentiation effect in an in vivo tumor growth model. Beatty et al. reported that α-eleostearic acid acts toward an increase in lipid peroxidation and ferroptosis of BC cells by promoting ACSL1 [48]. The molecular basis and drugs targeting iron toxicity and lipid peroxidation of ferroptosis in BC are summarized in Figure 1, and the current therapeutic approaches aiming to induce iron accumulation and lipid peroxidation are summarized in Table 2.

## 3. Inducing Ferroptosis by Inhibiting Antioxidant Defense

**Figure 2:**
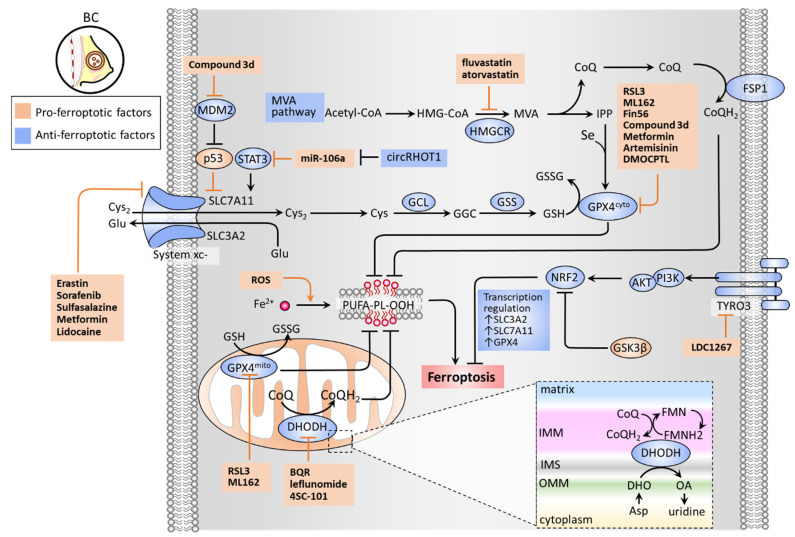
Antioxidant defense system that regulates ferroptosis. GSH-dependent pathways (GSH/GPX4 and MVA) and GSH-independent pathways (FSP1/CoQ10 and DHODH/CoQ10) constitute an antioxidant defense system. System xc− composed of SLC3A2 and SLC7A11 is a cystine/glutamate antiporter that imports Cys2 into cells with an equal amount of counter-transport of glutamate. The Cys_2_ is oxidized to cysteine (Cys), which contributes to the synthesis of GSH in a reaction catalyzed by glutamate-cysteine ligase (GCL) and glutathione synthetase (GSS). Cytosolic and mitochondrial GPX4 (GPX4^cyto^ and GPX4^mito^) eliminates ROS by converting GSH to GSSG. The MVA pathway requires HMGCR as a critical rate-limiting enzyme to generate IPP and CoQ10. IPP is involved in the synthesis of GPX4 in the presence of Se, while CoQ10 plays an important role in suppressing lipid peroxidation. FSP1 repairs lipid peroxidation on the plasma membrane by converting CoQ10 to CoQH_2_. By contrast, DHODH generates CoQH2 by reducing CoQ10 through a uridine-synthesizing redox reaction that catalyzes DHO to OA to exert a protective effect on lipid peroxidation at the mitochondrial membrane. TYRO3 acts toward anti-ferroptosis by the PI3K/AKT/NRF2 axis to bolster the activity of GSH/GPX4. Abbreviations: BQR, brequinar sodium; circRHOT1, circular RNA RHOT1; CoQ10, coenzyme Q10; Cys, cysteine; Cys2, cystine; DHO, dihydroorotate; DHODH, dihydroorotate dehydrogenase; FSP1, ferroptosis suppressor protein 1; GCL, glutamate-cysteine ligase; GPX4, glutathione peroxidase 4; GSH, glutathione; GSK3B, glycogen synthase kinase 3 beta; GSS, glutathione synthetase; HMGCR, HMG-CoA reductase; IMM, inner membrane of mitochondria; IMS, intermembrane space; IPP, isopentenyl-pyrophosphate; MDM2, mouse double minute 2; MVA, mevalonate; NRF2, nuclear factor, erythroid 2-like 2; OA, orotate; ROS, reactive oxygen species; OMM, outer membrane of mitochondria; Se, selenium; SLC3A2, solute carrier family 3 member 2; SLC7A11, solute carrier family 7 member 11; STAT3, signal transducer and activator of transcription 3; TYRO3, tyrosine-protein kinase receptor 3.

There are several classes of antioxidant pathways that counteract ferroptosis. These include the glutathione (GSH)-dependent phospholipid hydroperoxidase glutathione peroxidase 4 (GPX4) pathway in the cytosol (GPX4^cyto^) and mitochondria (GPX4^mito^), while the GSH-independent coenzyme Q10 (CoQ10, also known as ubiquinone) pathway is underpinned by ferroptosis suppressor protein 1 (FSP1, also known as apoptosis-inducing factor mitochondrial 2 (AIF-M2)) at the plasma membrane (FSP1-CoQ10 axis) and dihydroorotate dehydrogenase (DHODH) in the mitochondrial inner membrane (DHODH-CoQ10 axis) (Figure 2) [89,90]. 

The synthesis of GSH relies mainly on the import of cystine (Cys_2_). System xc^−^ is a cystine/glutamate antiporter widely distributed in phospholipid bilayers, acting to import Cys_2_ into cells with a 1:1 counter-transport of glutamate [6,30] and preserve the homeostasis of the antioxidant system in cells. System xc^−^ is a heterodimer composed of two subunits: solute carrier family 7 member 11 (SLC7A11) and solute carrier family 3 member 2 (SLC3A2). The Cys_2_ taken up into cells can then be oxidized to cysteine (Cys), which is required for the synthesis of GSH in a reaction catalyzed by glutamate-cysteine ligase (GCL) and glutathione synthetase (GSS) [31]. GSH functions to reduce reactive oxygen species (ROS) and reactive nitrogen under the action of glutathione peroxidases (GPXs). Among the GPX family, GPX4 plays a critical role in regulating the occurrence of ferroptosis. GPX4 can convert GSH into oxidized glutathione (GSSG) and reduce cytotoxic lipid peroxides (L-OOH) to the corresponding alcohols (L-OH), thereby inhibiting the formation of lipid peroxides (Figure 2). RSL3 and ML162 can serve as GPX4 inhibitors to promote cell ferroptosis [90]. The mevalonate (MVA) pathway counteracts ferroptosis by generating anti-ferroptotic biomolecules, including isopentenyl-pyrophosphate (IPP) and CoQ10. The synthetic processes of the two molecules require a rate-limiting enzyme, HMG-CoA reductase (HMGCR), which is also an inhibitory target of statins (a class of cholesterol-lowering drugs) [35]. IPP acts to stabilize selenocysteine tRNA, which is required for the synthesis of GPX4 [91]. Concerning the GPX4-independent CoQ10 pathway, Bersuker et al. first identified that FSP-1, a flavoprotein formerly known as AIF-M2 (apoptosis-inducing factor mitochondrial 2), exhibits a protective effect against ferroptosis, as induced by GPX4 deletion [92]. At the plasma membrane, FSP-1 acts as an oxidoreductase that reduces CoQ10 to generate CoQH2 (also known as ubiquinol) which can repair lipid peroxides [93]. More recently, Mao et al. reported that the mitochondrial enzyme dihydroorotate dehydrogenase (DHODH) acts to coordinate with GPX4^mito^ to prevent ferroptosis by detoxifying accumulated lipid peroxides in mitochondria [89]. DHODH has been recognized as an iron-containing flavin-dependent enzyme, and is involved in the de novo synthesis of pyrimidines in mitochondria [94]. Further study revealed that DHODH generates CoQH2 by reducing CoQ10 through a uridine-synthesizing redox reaction that catalyzed dihydroorotate to orotate [89]. DHODH inhibitors have previously been used in the treatment of autoimmune diseases, such as multiple sclerosis and rheumatoid arthritis [90]. The findings of Mao et al. regarding the role of DHODH in ferroptosis could exploit the synthetic lethal (SL) concept, by which DHODH inhibitors exert an anticancer effect in GPX4^low^ cells; meanwhile, the combination of DHODH inhibitors with sulfasalazine (an inhibitor of system xc^−^) can be applied to GPX^high^ cells [89].

Wen et al. reported that an active compound from the medicinal herbal licorice, 18-β-glycyrrhetinic acid, could induce ferroptosis in TNBC cells by downregulating the expression of SLC7A11 of system xc-, the GSH level, the GPX activity, and upregulating the ROS and lipid peroxidation [54]. Sun et al. reported that lidocaine exerts a pro-ferroptosis effect in BC and ovarian cancer by targeting the miR-382-5p/SLC7A11 axis [51]. Zhang et al. revealed that microRNA(miR)-106a-5p promotes ferroptosis via the suppression of the signal transducer and activator of transcription 3 (STAT3), whereby SLC7A11 levels are downregulated in BC cells [52]. Circular RNA RHOT1 (circRHOT1) has been identified as a ferroptosis inhibitor by sponging miR-106-5a [52]. Liu et al. revealed a nitroisoxazole-containing compound (3d) that can effectively induce ferroptosis of MCF-7 both in vitro and in vivo [60]. As a GPX4/mouse double minute 2 (MDM2) dual inhibitor, 3d presents dual inhibitory activities with the suppression of GPX4 levels and MDM2-mediated degradation of p53 [60]. Wu et al. demonstrated that overexpression of glycogen synthase kinase-3β (GSK-3β) sensitizes erastin-induced ferroptosis by inhibition of a nuclear factor, erythroid 2-like 2 (NFE2L2, also known as NRF2), thereby downregulating GPX4 levels [56]. It is worth noting that upregulation of NRF2 can lead to ferroptosis resistance. Qiao et al. demonstrated that upregulated NRF2 contributed to BET inhibitor-induced ferroptosis in BC cancer cells [95]. siRNA-based knockdown of nuclear receptor subfamily 5 group A member 2 (NR5A2) and nuclear receptor coactivator 3 (NCOA3) caused a decrease in NRF2 expression levels, thereby counteracting BET inhibitor-induced ferroptosis. Verma et al. demonstrated a high-throughput screen reliant on the concept of SL which reported that a combination of BET inhibitor JQ1 and proteasome inhibitor bortezomib (BTZ) (JQ1+BTZ) considerably induced TNBC ferroptosis by inhibiting GPX4 expression levels [55]. Ding et al. demonstrated that DMOCPTL, a derivative of the natural product parthenolide, exhibited a pro-ferroptosis effect on TNBC cell growth by inducing GPX4 ubiquitination [59]. Lee et al. reported that erastin induces TNBC cell ferroptosis by inhibiting system xc^−^ and depleting GPX4 levels, despite the resistance against oxidative stress [50]. Jiang et al. reported that tyrosine-protein kinase receptor 3 (TYRO3) plays a negative role on tumor ferroptosis, and induces resistance to anti-PD1/PD-L1 treatment [32]. TYRO3 overexpression activates the PI3K/AKT pathway to increase NRF2 transcriptional activity that is responsible for the transcription of ferroptosis-inhibitory genes, including SLC3A2, SLC7A11, FTL, FTH1, GPX4, SLC40A1, and biliverdin reductase A/B (BLVRA/B) [32]. Further study showed that the TYRO3 inhibitor, LDC1267, can elicit ferroptosis and potentiate immunotherapy in a 4T1 BC cell inoculation mouse model [32]. Song et al. developed acidity-activatable dynamic nanoparticles encapsulating RSL-3 to target GPX4, unveiling a strategy for ferroptosis-inducing tumor specific delivery. This nanoparticle approach acts to potentiate immunotherapy by recruiting tumor-infiltrating T lymphocytes for interferon gamma (IFNγ) secretion [57]. Hou et al. demonstrated that metformin induces TNBC cell ferroptosis via an upregulation of miR-324-3p, by which GPX4 expression is inhibited by targeting its 3′UTR [58]. In addition, Yang et al. revealed that antidiabetic metformin acts as an anti-BC agent by reducing the protein stability of SLC7A11 of system xc-, increasing intracellular Fe^2+^ and lipid ROS levels [53]. Furthermore, the combination of metformin with sulfasalazine, the system xc- inhibitor, can cause a potentiation effect to induce ferroptosis [53]. Concerning the blocking of the MVA pathway as a targeting strategy, two clinical trials involving BC patients have indicated that fluvastatin and atorvastatin might have antiproliferative effects in tumors overexpressing HMGCR [63,64]. Hubackova et al. reported that the DHODH inhibitor leflunomide exerts a potentiation effect on inducing TNBC cell death in vitro, and tumor growth in vivo, when combined with a checkpoint kinase 1 (Chk1) inhibitor [61]. Mohama Fairus et al. demonstrated that DHODH inhibitors, including brequinar sodium (BQR), leflunomide, and 4SC-101 provoked ROS generation and ATP depletion by a p53-mediated route, thereby suppressing BC cell proliferation [62]. The molecular basis and drugs targeting the antioxidant defense systems of ferroptosis in BC, including GPX4/GSH, MVA, FSP1-CoQ10, and DHODH-CoQ10, are illustrated in Figure 2. The current therapeutic approaches for targeting these pathways are summarized in Table 2.

## 4. Conclusions

There exist several tumor subtypes of BC, which are highly heterogenous malignancies, and considered particularly challenging to treat due to their resilience to standard therapeutic agents and high probability of recurrence. The past five years have seen a growing number of studies focused on the role of ferroptosis in BC. These investigations have further clarified our understanding of ferroptosis, which involves the integration of a highly organized system that regulates iron metabolism, lipid peroxidation, and anti-oxidant defense, relying on specialized mechanisms in the plasma membrane, mitochondria, and cytosol. The accumulated knowledge has been exploited to further improve the prevention and treatment of this disease. For instance, mounting preclinical evidence indicates that the induction of ferroptosis may be an effective therapeutic strategy to prevent acquired resistance to several cancer therapies, and to exert a potentiation effect on immunotherapy [32,42,49]. More importantly, clinical trials that apply ferroptosis-inducing agents to patients with BC are ongoing. As such, it is inevitable that continuing research in this field will further elucidate the physiological and pathological roles of ferroptosis, leading to the development of translational anticancer strategies. Research into biomarkers to precisely trace ferroptosis in patients with cancer, and the development and subsequent application of novel ferroptosis-based therapies will be of critical importance in the next few years.
